# Supplementary material for: The causal role of circulating inflammatory markers in osteoporosis: a bidirectional Mendelian randomized study
Source: Front Immunol. 2024 Jul 18;15:1412298. doi: 10.3389/fimmu.2024.1412298 (PMC11291241; doi:10.3389/fimmu.2024.1412298)
Supplement: Supplementary file 1 [file DataSheet_1.zip › supplementary material/CIM-OSTEOPOROSIS /GCST90274826.csv_leave_one_out.pdf]

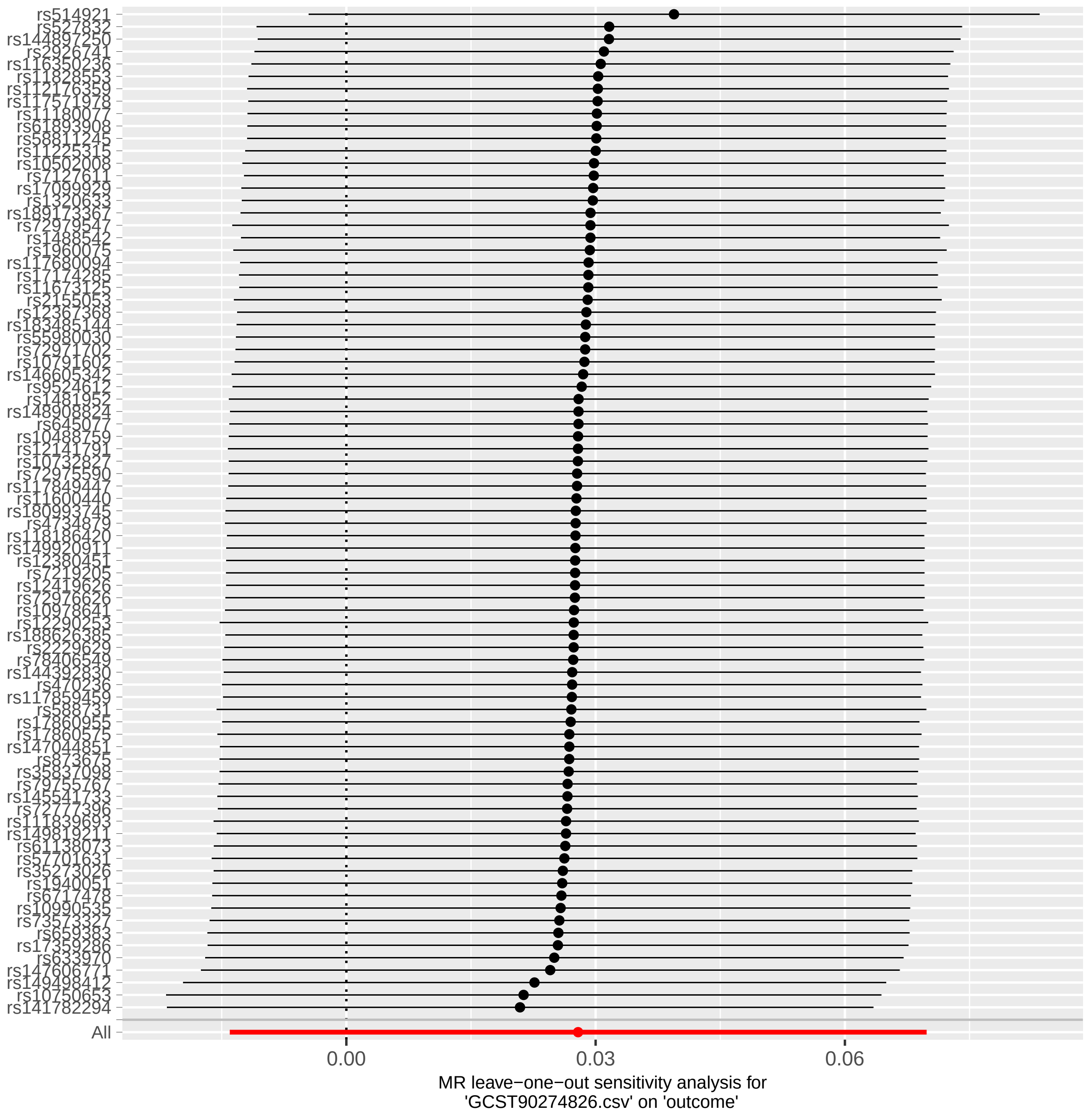
rs514921
rs527832
rs144897250
rs2926741
rs116350236
rs11828553
rs112176359
rs117571978
rs11180077
rs61893908
rs58811245
rs11225315
rs10502008
rs7127611
rs17099929
rs1320633
rs189173367
rs72979547
rs1488542
rs1960075
rs117680094
rs17174285
rs11673125
rs2155053
rs12367368
rs183485144
rs55980030
rs72971702
rs10791602
rs146605342
rs9524612
rs1481952
rs148908824
rs645077
rs10488759
rs12141791
rs10732827
rs72975590
rs117849447
rs11600440
rs180993745
rs4734879
rs118186420
rs149920911
rs12380451
rs7219205
rs12419626
rs72976626
rs10978641
rs12290253
rs188626385
rs2229629
rs78406549
rs144392830
rs470236
rs117859459
rs588731
rs17860955
rs17860575
rs147044851
rs873675
rs35837098
rs79755767
rs145541733
rs72777396
rs111839693
rs149819211
rs61138073
rs57701631
rs35273026
rs1940051
rs6717478
rs10990535
rs73573327
rs659383
rs17359286
rs633970
rs147606771
rs149498412
rs10750653
rs141782294
All
0.00
0.03
0.06
MR leave−one−out sensitivity analysis for
'GCST90274826.csv' on 'outcome'
